# One-Pot Synthesis of Star Copolymers by the Combination of Metal-Free ATRP and ROP Processes

**DOI:** 10.3390/polym11101577

**Published:** 2019-09-27

**Authors:** Gorkem Yilmaz

**Affiliations:** Department of Chemistry, Istanbul Technical University, Maslak, 34469 Istanbul, Turkey; gorkemyilmaz@itu.edu.tr

**Keywords:** photochemical processes, metal-free polymerization, controlled/living radical polymerization, atom transfer radical polymerization, ring-opening polymerization

## Abstract

A completely metal-free strategy is demonstrated for the preparation of star copolymers by combining atom transfer radical polymerization (ATRP) and ring-opening polymerization (ROP) for the syntheses of block copolymers. These two different metal-free controlled/living polymerizations are simultaneously realized in one reaction medium in an orthogonal manner. For this purpose, a specific core with functional groups capable of initiating both polymerization types is synthesized. Next, vinyl and lactone monomers are simultaneously polymerized under visible light irradiation using specific catalysts. Spectral and chromatographic evidence demonstrates the success of the strategy as star copolymers are synthesized with controlled molecular weights and narrow distributions.

## 1. Introduction

**Scheme 1:**
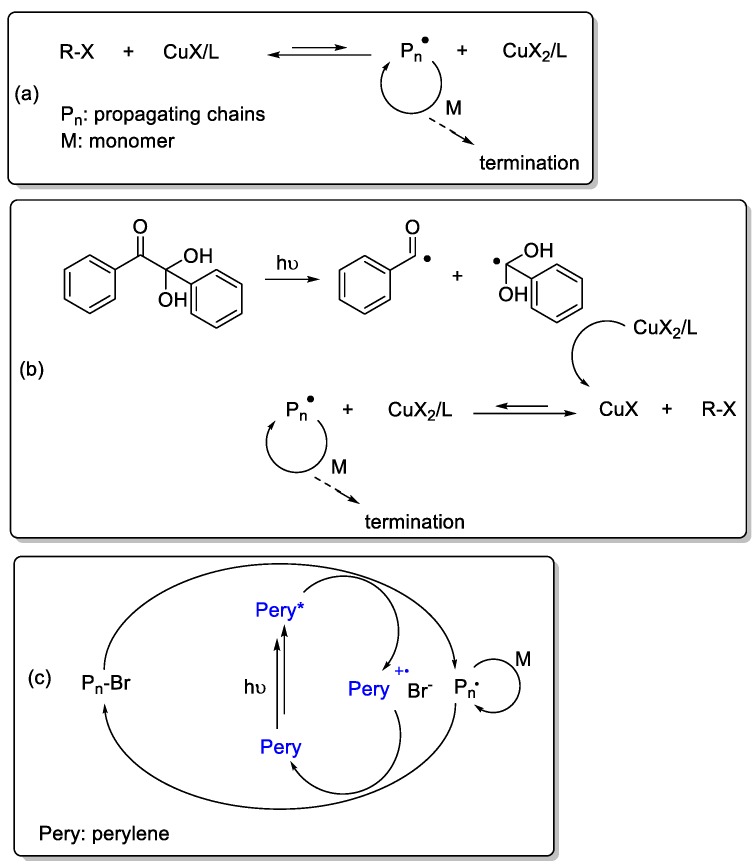
General representation of conventional atom transfer radical polymerization (ATRP) (**a**), photoinduced ATRP using 2,2-dimethoxy-2-phenylacetophenone (**b**) and photoinduced metal-free ATRP using perylene (c).

Synthesis of complex molecular architectures with well-defined structures and controlled molecular weight characteristics has been an attractive research area in the last decade [1,2]. The discovery of controlled polymerization (CP) methods, as well as highly efficient click reactions, enabled the preparation of various types of polymers in a fast and efficient manner [3,4,5,6,7,8,9,10,11]. Among the CP techniques applied, atom transfer radical polymerization (ATRP) has been the most widely studied strategy, as it is applicable to a wide range of vinyl monomers and provides the syntheses of halide-chain-end polymers with narrow molecular weight distributions [12]. In general, it requires large amounts of low oxidation state copper complexes (CuX/L) for the polymerization to occur (Scheme 1a). The major drawbacks of utilizing conventional ATRP is the vulnerability of the catalyst in oxidative conditions, requiring a high catalyst load, which should be removed after polymerization. This is especially important for the preparation of polymers, which will be used for bioapplications. For troubleshooting, both chemical and photochemical protocols have been suggested, which can realize ATRP under low metal catalyst concentrations. Typically, CuX_2_/L complexes are used, which are simultaneously reduced either with the help of specific compounds or by light-induced processes. Hydrazine and phenol are some of these reducing agents, which reduce CuX_2_ to CuX in the reaction media that mediate the ATRP process [13,14]. For photochemical processes, both direct and indirect pathways should be considered. The direct pathway considers the reduction of CuX_2_/L under light irradiation [15], whereas an additional photochemical compound is required in the indirect pathway [16,17,18,19]. Below is an example of indirect reduction mechanism by the aid of a radical initiator, namely 2,2-dimethoxy-2-phenylacetophenone (Scheme 1b). Notably, the light-induced processes are in an advanced state as they facilitate temporal and spatial control over the polymerization processes [20]. Therefore, they are also applied to other polymerization modes in addition to click processes [21,22,23,24,25]. 

Recent efforts have shown the possibility of conducting ATRP without the necessity of metal catalysts. In the presence of specific photochemical reagents, it was demonstrated that ATRP could be realized even in the absence of metal catalysts upon light irradiation [26]. Such photocatalysts are phenothiazines [27,28,29], dihydrophenazines [30], phenoxazines [31], perylene [32], pyrene [33], thienothiophenes [34], and certain dye/amine combinations [35,36,37]. Scheme 1c shows the typical mechanism when perylene is used as photocatalyst for such reactions. 

Ring-opening polymerization (ROP) is also a controlled/living process, which is widely used for the syntheses of polyesters. Traditionally, it requires an alcohol as an initiator and stannous octoate as a catalyst. Recent efforts have demonstrated that ROP can also be carried out under metal-free conditions [38,39]. It has been shown that phosphazene base (P_2_-*t*-Bu) catalyzed polymerization of ε-caprolactone (CL) polymerization proceeds smoothly under mild conditions to produce monodisperse poly(ε-caprolactone) (PCL). 

**Scheme 2:**
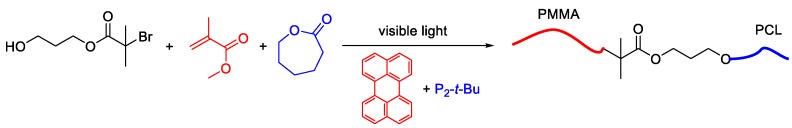
Combination of photoinduced metal-free ATRP and ring-opening polymerization (ROP) processes for block copolymer synthesis.

In addition to specific synthetic applications of these polymerization procedures, recently it has been shown that these two processes can be conducted concurrently in one reaction medium to afford block copolymers [40]. By using a dual initiator strategy, vinyl monomers and lactones have been simultaneously polymerized form the initiator core without affecting each other as shown below (Scheme 2). 

This way, one could prepare block copolymers in a one-shot manner under visible light irradiation, which would require multiple polymerization and purification steps if prepared in a step by step manner. Therefore, this synthetic method provides simple and environmentally-friendly conditions for the preparation of such complex macromolecular structures in short times.

Star copolymers display outstanding physicochemical properties, which cannot be attained by linear polymers [41,42]. Therefore, the synthesis of star copolymers is of high synthetic importance. They can be prepared by applying two distinctive approaches, namely arm-first and core-first approaches [43]. The former considers the use of a macroinitiator or a macromonomer to form the star copolymer with a crosslinked core. In the core-first approach, first, a functional core is prepared. Then the polymers are either grafted from the core or individually prepared and grafted onto the core by coupling reactions. 

Herein, a similar strategy has been demonstrated for the preparation of star copolymers by applying a core-first strategy. For this purpose, a specific core is designed and synthesized bearing functionalities capable of initiating ATRP and ROP. Next, both polymerizations have been carried out simultaneously using appropriate monomers under visible light irradiation.

## 2. Materials and Methods

### 2.1. Materials

Methyl methacrylate (MMA, 99%; Aldrich, Saint Louis, USA) and styrene were washed with NaOH and distilled over CaH_2_ prior to use. ε-caprolactone (ε-CL, 97%; Aldrich, Saimt Louis, USA), was also vacuum distilled over CaH_2_ and collected in molecular sieves before use. Tetrahydrofuran (THF; Aldrich HPLC grade) was dried on sodium wire under reflux in the presence of traces of benzophenone until a blue color persisted and was used directly after distillation. Phosphazene base (P_2_-*t*-Bu, ~2.0 M THF solution, Sigma-Aldrich) and perylene (98%, Sigma Aldrich, Saint Louis, USA) were used as received. 

### 2.2. Characterization

^1^H NMR of the intermediates and final polymers were recorded at room temperature at 500 MHz on an Agilent VNMRS 500 spectrometer (Santa Clara, USA). Molecular weights and polydispersities of the polymers were measured using gel permeation chromatography (GPC) employing an Agilent 1100 instrument equipped with a differential refractometer using THF as the eluent at a flow rate of 0.3 mL min^−1^ at 30 °C and polystyrene standards. 

### 2.3. Synthesis of (2,2,5-trimethyl-1,3-dioxan-5-yl)methyl 2-bromo-2-methylpropanoate

The core was synthesized in three steps, according to modified procedures reported in the literature. (2,2,5-Trimethyl-1,3-dioxan-5-yl) methanol, which was synthesized following the procedure by Jia et al. [44]. (1.5 g, 1 eq) was taken into a two-necked round bottom flask equipped with a magnetic stirrer and dissolved in CH_2_Cl_2_ (15 mL). Then, TEA (1.4 mL, 1.2 eq) was added to the mixture, which was then cooled to 0 °C. Next, α-bromoisobutyryl bromide (1.2 g, 1.2 eq) in 5 mL of CH_2_Cl_2_ was slowly added to the reaction mixture using an addition funnel. The reaction mixture was left to stir at room temperature for overnight. The resulting mixture was filtered off and the solvent was evaporated. The product was purified by column chromatography over silica using ethyl acetate/petroleum spirit (*v*/*v*: 1/2). Yield: 71%, ^1^H NMR (CDCl_3_), δ: 4.28 (s, 2H, –CO–O–CH_2_–C), 3.65 (q, 4H, –O–CH_2_–C), 1.98 (s, 6H, methyl protons), 1.40 (3H, methyl protons), 0.94 (s, 3H, methyl protons).

### 2.4. Synthesis of 3-Hydroxy-2-(hydroxymethyl)-2-methylpropyl 2-bromo-2-methylpropanoate (the Core)

(2,2,5-Trimethyl-1,3-dioxan-5-yl)methyl 2-bromo-2-methylpropanoate (1 g, 0.32 mol) was dissolved in 30 mL of methanol, and HCl (0.3 mL) was added to the solution. The solution was refluxed overnight. After completion of the reaction, the solvent was evaporated and the white crystals were dried under reduced pressure. Yield: 94%, ^1^H NMR (CDCl_3_), δ: 4.28 (s, 2H, –CO–O–CH_2_–C), 3.63 (d, 4H, –C–CH_2_–OH), 1.95 (s, 6H, methyl protons), 0.92 (s, 3H, methyl protons).

### 2.5. General Procedure for Block Copolymer Synthesis: Simultaneous Metal-Free ATRP and ROP Processes Under Visible Light

In a typical experiment, the following chemicals were taken in a dry Schlenk tube containing dry THF under nitrogen atmosphere: [MMA] or [St]/[Perylene]/[core]: 200/2/1, [ε-CL]/[P_2_-*t*-Bu]/[core]: 200/3/1, V_MMA_ = V_THF_ = 1 mL. The tube was exposed to visible light irradiation (λ = 400–500 nm, light intensity: 40 mW cm^−2^) for 2 h and the mixture was precipitated in methanol. The material was reprecipitated in methanol, filtered off, and dried under vacuum. 

## 3. Results and Discussion

**Scheme 3:**
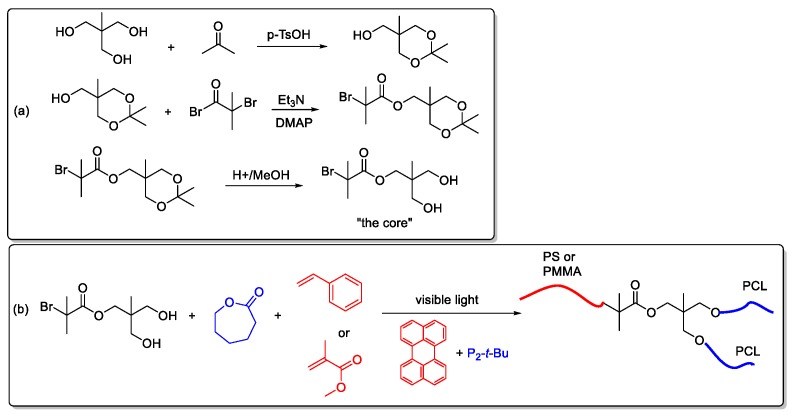
Syntheses of the core (**a**) and star copolymers (**b**).

In order to prepare miktoarm star copolymers using a combination of photo-induced metal-free ATRP and ROP processes, first, a core with appropriate functional groups for both polymerizations was synthesized according to modified procedures reported in the literature [45,46]. For this purpose, first 2-(hydroxymethyl)-2-methylpropane-1,3-diol was reacted with acetone, which was then subjected to an esterification reaction to attain bromide functionalities. Then the protecting acetone group was broken to give the targeted core structure as shown below (Scheme 3a). After it has been synthesized, the functional groups of the core were used as initiating sites for both ATRP and ROP using perylene and phosphazene base as catalysts. Methyl methacrylate (MMA) or styrene (S) were used together with CL to obtain AB_2_ type star copolymers, namely PS-(PCL)_2_ and PMMA-(PCL)_2_ (Scheme 3b).

**Figure 1:**
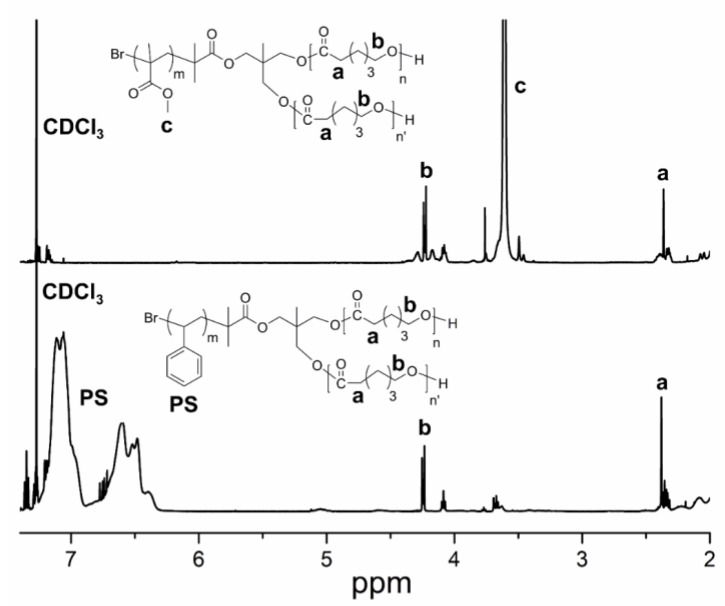
^1^H NMR spectra of PMMA-(PCL)_2_ and PS-(PCL)_2_.

The structures of both of the star copolymers, were investigated by ^1^H NMR analysis. Clearly, ^1^H NMR spectra of the star copolymers exhibit the characteristic signals of the corresponding segments. Aromatic signals of the PS segment appear between 6.5–7.2 ppm, whereas the characteristic peaks of PCL are observable at 2.4 and 4.2 ppm. Similarly, the ^1^H NMR spectrum of PMMA-(PCL)_2_ exhibits the characteristic ester protons of the PMMA segment at 3.6 ppm, together with the peaks of the PCL fragments (Figure 1).

**Figure 2:**
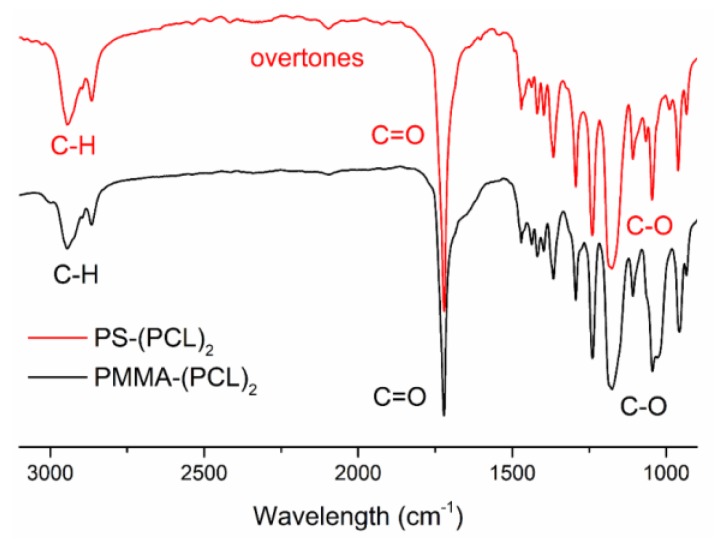
FT-IR spectra of PMMA-(PCL)_2_ and PS-(PCL)_2_.

The structures of the polymers were further confirmed by FT-IR analyses (Figure 2). The FT-IR spectrum of PS-(PCL)_2_ displays the characteristic stretching bands around 1600 and 1730 cm^−1^, which correspond to the C=C double bonds and ester carbonyl. The overtones around 2000 cm^−1^ also show the presence of the PS segment. The FT-IR spectrum of PMMA-(PCL)_2_ also shows an intense peak at 1730 cm^−1^, which belongs to the ester functionalities of PMMA and PCL segments. 

**Figure 3:**
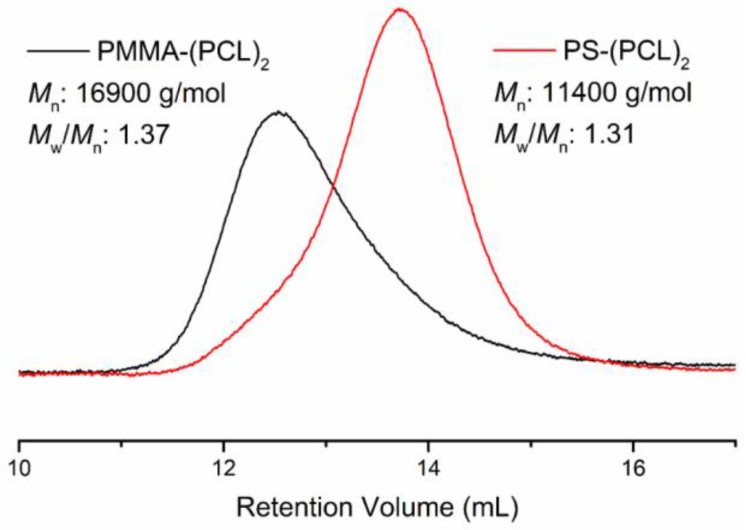
Gel permeation chromatography (GPC)traces of PMMA-(PCL)_2_ and PS-(PCL)_2_ star copolymers.

The molecular weight characteristics of the star copolymers were investigated by GPC analyses as shown below (Figure 3). Clearly, both polymers exhibit a unimodal molecular weight distribution, which excludes the possibility of the presence of unreacted polymer segments or occurrence of any side reactions. 

**Figure 4:**
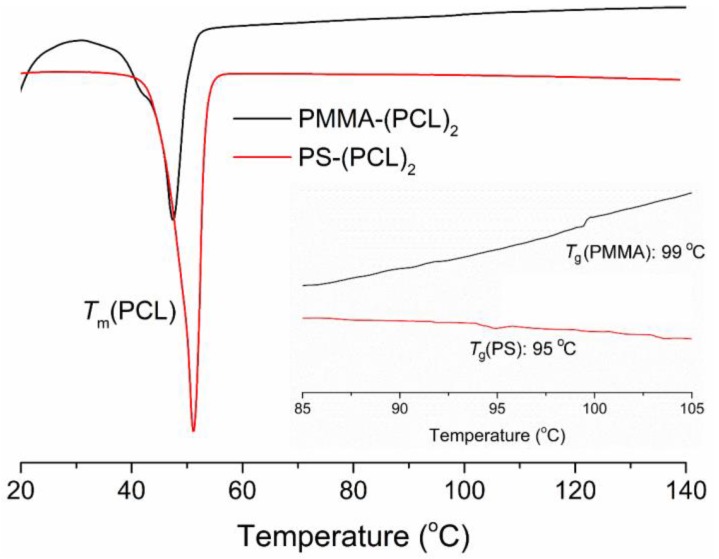
DSC thermograms of PMMA-(PCL)_2_ and PS-(PCL)_2_ star copolymers.

The thermal properties of the star copolymers were also investigated (Figure 4). The DSC thermograms of both polymers were analyzed and, in each case, the melting point (*T*_m_) of the PCL segment can be easily distinguished around 43–45 °C. The glass transition temperatures (*T*_g_) of PS and PMMA segments were observed at 95 and 99 °C, respectively. 

## 4. Conclusions

In conclusion, the preparation of star copolymers using structurally different monomers can be simply and efficiently realized in a very short time. By the combination of completely metal-free approaches namely, photo-induced ATRP and ROP processes, star copolymers can be synthesized in a simultaneous manner. Using this method, the reaction and purification steps are reduced to a single step, which is a meaningful advantage in terms of economical and eco-friendly concerns. This straightforward strategy is expected to be applied for the synthesis of other complex macromolecular structures or for other monomer compositions. Notably, the metal-free feature of the approach provides polymers without metal contamination, which can be of high priority for templates used for bioapplications. Further studies in this line are now in progress.
